# ERα increases expression and interacts with TERT in cataractous canine lens epithelial cells

**Published:** 2009-11-09

**Authors:** Carmen M.H. Colitz, Yasuro Sugimoto, Ping Lu, Curtis Andrew Barden, Jennifer Thomas-Ahner, Heather Lynn Chandler

**Affiliations:** 1 Veterinary Clinical Sciences, Ohio State University, Columbus, OH; 2 Divison of Medicinal Chemistry and Pharmacognosy, Ohio State University, Columbus, OH; 3 Department of Pathology, Ohio State University, Columbus, OH

## Abstract

**Purpose:**

Estrogen receptor alpha (ERα) expression has previously been evaluated in lens epithelial cells (LEC). However, its function in the lens has not been determined. One potential function may be its interaction with the catalytic subunit of telomerase (TERT), which is present in normal LEC and higher in LEC that have undergone epithelial to mesenchymal transition (EMT). ERα is known to play a role in EMT, a process that may also involve TERT.

**Methods:**

A commercially available transcription factor array was used to evaluate potential interactions between TERT and other proteins in normal and cataractous LEC. Based on these findings, ERα protein and mRNA expressions were measured using western blot analysis, immunohistochemical staining, and quantitative reverse transcription polymerase chain reaction (RT–PCR). Co-immunoprecipitation assays were used to evaluate the interaction of TERT with ERα as well as their phosphorylation in normal and cataractous LEC.

**Results:**

The transcription factor array suggested that TERT interacted with ERα via the estrogen response element (ERE) in cataractous LEC but not in normal LEC. Expression of ERα protein and mRNA increased in cataractous LEC compared with normal LEC. Co-immunoprecipitation assays confirmed the interaction of TERT with ERα in cataractous LEC while no interaction was found in normal LEC. LEC that have undergone EMT, e.g., cataracts, are rapidly proliferating and migrating along the posterior lens capsule.

**Conclusions:**

ERα is known to play a role in EMT, and our data suggests that TERT and phosphorylated protein kinase B (pAkt) may be involved in the regulation of this process in cataractous LEC.

## Introduction

Estrogen receptor alpha (ERα) belongs to a superfamily of transcription factors that mediate transcription in a ligand-dependent or -independent manner. The ligand-dependent manner requires estrogen while the ligand-independent manner is by means of second messenger signaling mechanisms [1,2]. Estrogen receptors (ER) have been detected in various ocular tissues including the ciliary body, meibomian glands, conjunctiva, lacrimal gland, and tarsal plate as well as in human, rat, and mouse lens epithelial cells (LEC) [3-5], though the exact role of ER in these tissues is not known. ERα has also been recently been shown to be involved in epithelial to mesenchymal transition (EMT) [6,7], a major component of normal wound healing [6,8,9].

Aberrant proliferation and posterior migration of the LEC are changes that occur in LEC during cataractogenesis and secondary cataract (also known as posterior capsule opacification [PCO]). These were previously referred to as fibrous metaplasia or pseudometaplastic changes [10]. These LEC have genotypic and phenotypic changes consistent with EMT and are thought to be a wound healing response of LEC in an attempt to repopulate the lens capsule [11-14]. Lenticular EMT results in the migration of the LEC onto the posterior lens capsule along with the production of aberrant extracellular matrix proteins, which result in subcapsular plaques. These changes are seen in advanced senile cataracts as well as in inherited, congenital, diabetic, traumatic, and ultraviolet radiation (UV)-induced cataracts and during PCO regardless of cause or species [15-19].

We have previously shown that telomerase expression is relatively low in normal LEC and increased in cataractous LEC [20], i.e., LEC that have undergone EMT. To evaluate telomerase regulation in these LEC, the present study compared normal and cataractous LEC using a transcription factor array assay, which evaluated proteins interacting with an estrogen response element (ERE). Results from this assay led us to evaluate differences in ERα mRNA and protein expressions between normal LEC and LEC that have undergone EMT. Finally, a probable interaction between telomerase reverse transcriptase (TERT) and ERα was found in LEC that had undergone EMT and was confirmed using co-immunoprecipitation. These findings further our understanding of telomerase regulation in normal LEC and those that have undergone EMT.

## Methods

### Samples

#### Normal lens and anterior lens capsules

Normal ocular tissues were obtained by enucleation from dogs in good general health with normal eyes that were euthanized at a local animal shelter for population control purposes. The animals were humanely euthanized, and the globes were collected within 1 h of death. Eyes were immediately placed in 2% betadine solution and then rinsed and immersed in 1× phosphate buffered saline solution (PBS, pH 7.2) until dissection, which was performed within 2 h of enucleation.

Excess conjunctiva and Tenon's capsule were excised from the perilimbal region, and a stab incision was made into the anterior chamber 1 mm posterior to the limbus. Anterior lens capsules with adherent LEC were excised from each lens for protein or RNA extraction and immediately frozen and stored at –70 °C until extraction. Whole lenses were gently excised from the zonular vitreal attachments and fixed in 10% neutral buffered formalin for immunohistochemical staining.

#### Cataractous anterior lens capsules

Anterior capsulorhexis specimens from dogs with naturally developing cataracts were obtained before routine phacoemulsification cataract extraction. Naturally occurring cataracts in canine patients are typically due to breed-related or inherited causes or secondary to diabetes mellitus [21]. Breed-related cataracts vary in presentation but often have diffuse cortical opacities, and classically, some breeds will initially have posterior subcapsular or posterior cortical polar cataracts that may or may not progress. Diabetic cataracts in dogs are rapidly occurring and encompass the entire cortex. Canine cataract patients undergo surgery much later than human cataract patients, therefore, the cataracts are in the late immature, mature, or hypermature stages. These stages of canine cataracts almost universally have LEC that have undergone EMT and posterior migration onto the posterior lens capsule [22]. Samples chosen for this study had obvious subcapsular plaques or diffuse LEC proliferation onto the posterior lens capsule evident at the time of phacoemulsification. Patients with only nuclear cataracts were not included in this study as surgery is rarely performed on this type of cataract.

Fixed samples were obtained by continuous curvilinear capsulorhexis and either immediately placed in 10% neutral buffered formalin or snap frozen and stored at –70 °C until protein or RNA extraction. Samples were paraffin embedded, sectioned, stained with hematoxylin and eosin (H&E), and then examined by light microscopy. For immunohistochemical staining, samples were sectioned onto charged slides (ProbeOn Plus; Fisher Scientific, Pittsburgh, PA).

### Protein extraction

Whole cell protein was extracted from frozen normal and cataractous samples following the manufacturer’s instructions (Chemicon International, Temecula, CA) with minor adaptations for our tissue as previously described [20].

### Transcription factor array

The TranSignal™ TF-TF Interaction Array I from Panomics (Fremont, CA) was used to screen for 54 transcription factor (TF)-protein interactions. Briefly, nuclear protein was extracted from two normal and four cataractous anterior lens capsules. The normal lens capsule samples were approximately seven years of age. Of the cataractous samples, two of the inherited samples and one of the diabetic samples were seven years old. The other diabetic sample was nine years of age. The protein was quantified by Bradford assay. Each of the samples was evaluated in two independent experiments with a negative control (sample omitted) included each time. Each sample was incubated with the biotin-labeled, double-stranded oligonucleotide probes provided, allowing the TF cis-elements to bind the TERT protein in the sample extracts. An immunoprecipitation was then performed using an antibody to TERT (Calbiochem, San Diego, CA) to pull out the TF cis-elements interacting with TERT. Non-specific binding was then washed away. The cis-elements were bound to TERT, and the anti-TERT antibody were then eluted and hybridized to the TranSignal Array membrane (one membrane per sample and a negative control; Panomics, Fremont, CA) using the kit’s horse-radish peroxidase (HRP)-based chemiluminescent detection system. The membranes were spotted with 54 different TF consensus sequences, and direct comparisons of spot intensity were made between the two sample blots using an AlphaImager (AlphaEaseFC software, Version 3.0; Alpha Innotech, San Leandro, CA) to identify differences in TF-TF binding element interactions. Each blot has positive controls incorporated along the bottom and side. In addition, each oligonucleotide corresponding to the TF binding site was spotted in duplicate and then again in duplicate at a dilution of 1:10 immediately below the full strength TF spots. Once scanned, each of the spot pairs was averaged, and statistical analyses were only performed on data from ERE, MRE (mineralocorticoid response element), p53 (tumor suppressor gene, p53), and Sp1 (transcriptional activator that regulates TERT, the catalytic subunit of telomerase) [23]. To control for inter-experiment differences, four blots underwent hybridization under identical conditions each time, and then all four membranes were exposed to the same piece of film. Each film was then scanned, and the positive control spots along the bottom and side of the membrane were used to normalize the test spots. Each pair of spots in the control rows that was directly to the right and to the bottom of the spots being analyzed were averaged and used to determine the value for each TF.

### Immunostaining for ERα and ERβ in normal and cataractous LEC

Standard avidin-biotin complex (ABC) immunohistochemical staining was performed as previously described [22]. Primary antibody, anti-ERα or anti-ERß (Santa Cruz Biotechnology Inc., Santa Cruz, CA), were diluted in antibody diluent (DAKO, Carpinteria, CA) at a dilution of 1:250. Stained samples were visualized by light microscopy and photographed (Olympus, Melville, NY). Ten normal and ten cataractous canine anterior lens capsules were used. Two separate canine mammary carcinoma samples were used as positive controls for ERα and ERß. In addition, murine normal skin and murine skin with ultraviolet-induced squamous cell carcinoma were also used as positive controls for ERß. The negative controls omitted the primary antibody. Experiments were repeated three times for each sample and antibody.

### Western blot analysis of ERα

Western blot analysis, performed as previously described [24], demonstrated the specificity of the anti-ERα antibody used in immunostaining experiments and was used as a semi-quantitative method to compare normal and cataractous anterior lens capsule samples. Primary antibodies used were anti-ERα antibody (1:1,500; Santa Cruz Biotechnology Inc) and anti-β-actin antibody (1:5,000; Sigma-Aldrich, St. Louis, MO). Protein signals were detected by chemiluminescence (Femto Western Blotting System; Pierce Biotechnology, Rockford, IL) using Kodak X-OMAT AR film (Eastman Kodak Co., Rochester, NY). Membranes were stripped (Restore stripping solution; Pierce Biotechnology), and the technique was repeated using the anti-β-actin antibody to control for gel loading. Kodak 1D Image Analysis Software (Kodak Molecular Imaging, New Haven, CT) was used to obtain densitometry readings for all western blots. Statistical analysis was performed with Prism software (GraphPad Prism® version 4; GraphPad Software, La Jolla, CA) using a Student *t*-test with Wilcoxon signed rank test. A p value less than 0.05 was considered statistically significant. Control samples for anti-ERα antibody included whole protein extract from a canine mast cell tumor and a canine mammary carcinoma, which both overexpress ERα.

### Quantitative RT-PCR for *ERα* in normal and cataractous LEC

RNA was extracted from normal and cataractous anterior lens capsule samples according to the suggested protocol using Absolutely RNA Microprep Kit (Stratagene, La Jolla, CA). The ImPromII Reverse Transcriptase kit (Promega, Madison, WI) was used to synthesize the first strand cDNA. Quantitative reverse transcription polymerase chain reaction (RT–PCR) was performed using the Mx3000p Multiplex Quantitation System (Stratagene) as follows: 95 °C for 15 min, then 40 cycles at 94 °C for 30 s, 60 °C for 30 s, and 72 °C for 30 s, using QuantiTect SYBR Green PCR kit (Stratagene). Primers to amplify sequences of *ERα* were designed based on previously published sequence data (GenBank AJ313195). Primers to amplify the sequence of hypoxanthine phosphoribosyl transferase (*HPRT*; housekeeping control) were based on previously published sequence data (NM_001003357). Primers employed were ERα/F823: 5'-GGG GAG GGC AGG AAT GAA GT-3′, ERα/R934: 5′-AGG GAC AAG GCT GGG CTG TT-3′, HPRT Forward: 5′-TGA CAC TGG TAA AAC AAT G-3′, and HPRT Reverse: 5′-GGT CCT TTT CAC CAG CAA GCT-3'.

All samples were run in duplicate three separate times. The threshold cycle value was calculated for each sample by the instrument software (Mx3000P real-time PCR machine; Stratagene, La Jolla, CA). The relative amount of *ERα* and *HPRT* mRNA was calculated using the LinRegPCR software (JM Ruijter, SvdVelden, A Ilgun, Amsterdam, the Netherlands) as previously described [25]. The results were expressed as the ratio of the target gene, *ERα*, to the HPRT housekeeping gene. Statistical analysis was performed with Prism software (GraphPad Prism® version 4) using a Student *t*-test with Wilcoxon signed rank test. A p value less than 0.05 was considered statistically significant. 

### Immunoprecipitation and immunoblotting of ERα and TERT

A total of eight normal and 18 cataractous canine anterior lens capsule samples were evaluated. Two kits were used to perform this experiment separately to ensure results were consistent. The Seize Classic Mammalian Immunoprecipitation kit and the Seize Mammalian Immunoprecipitation Plate-based kit (both from Pierce Biotechnology) were used following the manufacturer’s instructions. Briefly, the initial step coupled the appropriate affinity purified antibody (anti-ERα antibody; Santa Cruz Biotechnology Inc.) to the AminoLink Plus Coupling gel (ThermoFisher Scientific Inc., Rockford, IL). Five micrograms of protein were immunoprecipitated to the antibody coupled gel. Similar to the Classic kit, the plate-based kit’s initial step bound the capture antibody (anti-ERα; Santa Cruz Biotechnology Inc.) to the protein G coated wells. Protein samples (10 µg) were then immunoprecipitated, and following appropriate incubation and washes, the antigen:antibody complex was eluted. Controls for the plate-based Seize kit included a well without either antigen or antibody. Elutions from both kits were applied to a SDS–PAGE gel for electrophoresis and immunoblotting with anti-TERT antibody (TERT L-20; Santa Cruz Biotechnology Inc.). To ensure that results were consistent, the opposite was performed, i.e., capture with anti-TERT antibody and immunoblot with anti-ERα antibody. All experiments were performed in duplicate and repeated as well.

### Phosphorylation of ERα and TERT

Phosphorylation evaluation was performed using the Omni-Phos® Phosphorylation Assay Kit (Chemicon International, Inc, Temecula, CA) according to the manufacturer’s instructions. A co-immunoprecipitation approach using The Seize® Mammalian Immunoprecipitation Plate-based kit (Pierce Biotechnology) was used wherein either anti-ERα antibody (Santa Cruz Biotechnology Inc.) or anti-TERT was used to capture the protein present in each sample. Samples included normal canine anterior lens capsules (n=2), diabetic canine cataract samples (n=2), and breed-related canine cataract samples (n=2). Data shown is representative of both experiments. The captured protein was eluted, subjected to SDS–PAGE, and immunoblotted with the Omni-Phos® Blend, which includes phosphothreonine, phosphoserine, or phosphotyrosine antibodies according to the manufacturer’s directions. Blots were then stripped, and immunoblotting was performed with anti-pAkt antibody (1:1,000; Cell Signaling Technology®, Danvers, MA) under similar conditions described for western blot analysis. Secondary antibody used was donkey anti-mouse (1:8,000; Santa Cruz Biotechnology Inc.).

## Results

### Transcription factor array

**Figure 1:**
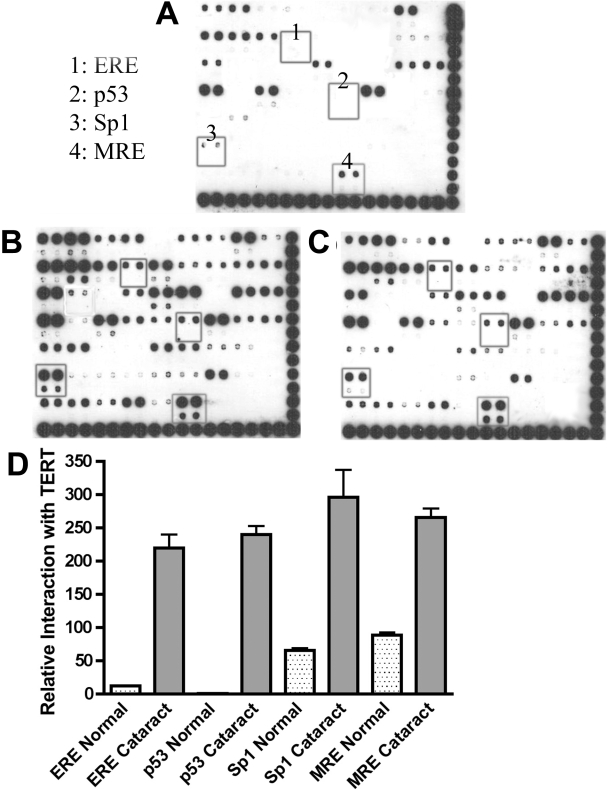
Transcription factor array comparing normal and cataractous LEC. **A-C**: The TransSignal™ (Panomics) TF-TF interaction array using one normal and two cataractous canine LEC samples. Nuclear extract was mixed with the TransSignal Probe mix, and immunoprecipitation was performed using a polyclonal antibody to TERT (Calbiochem, San Diego, CA). **A**: Normal sample from an approximately seven- to nine-year-old dog. **B**: Diabetic cataract from an approximately seven- to nine-year-old dog. **C**: Breed-related cataract from an approximately seven- to nine-year-old dog. Each TF is spotted on the blot in duplicate at the same concentration and then directly below those two spots at a 1:10 dilution in duplicate. Four of the TFs are boxed to show signal intensity differences: ERE (1), p53 (2), Sp1 (3), and MRE (4). It is evident that the normal sample shows less spot intensity of these TFs when compared to the cataract samples. **D**: Graph depiction of the four TFs boxed in **A**-**C** demonstrating interaction of TERT with ERE, p53, Sp1, and MRE. The cataractous samples had higher signal intensity than the normal samples. The normal samples showed some interaction of TERT with ERE, Sp1, and MRE. However, there was no p53 interaction with TERT. The blots shown are representative of repeated experiments. Error bars indicate standard error of the mean (SEM).

TFs that interacted with TERT in normal and cataractous LEC are shown in Figure 1. Four of these are highlighted in Figure 1 to demonstrate signal intensity differences. These are ERE, p53, Sp1, and MRE.

### Immunohistochemical staining for ERα and ERß in normal and cataractous LEC

**Figure 2:**
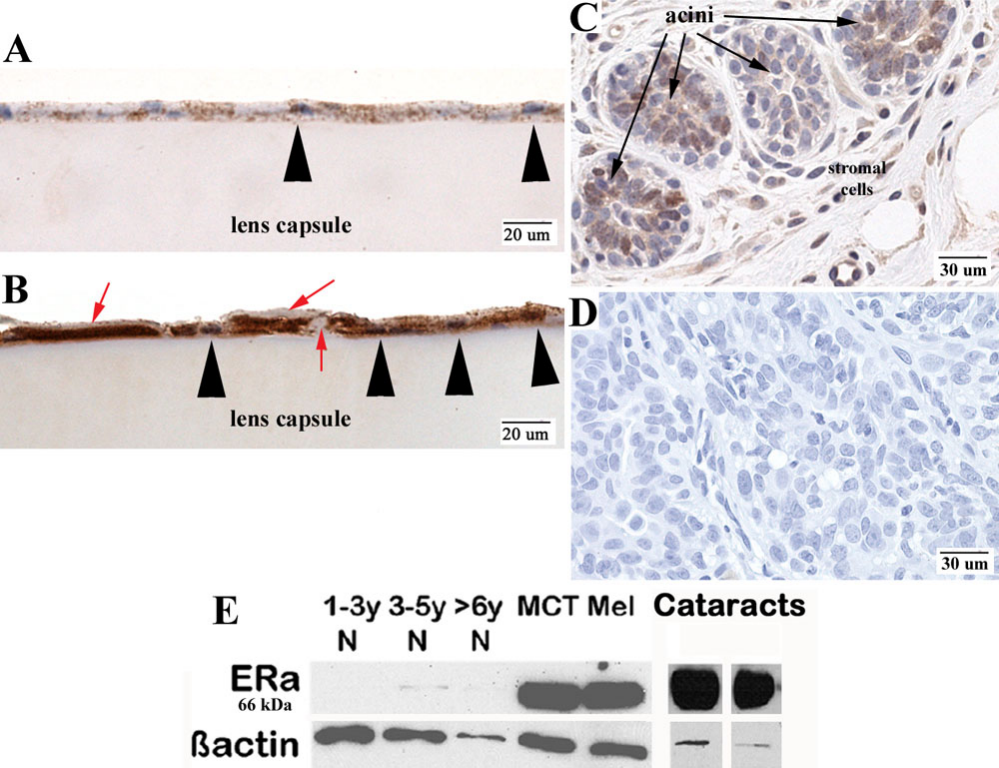
Protein expression of ERα in normal and cataractous LEC. Representative results are shown of immunohistochemical staining with anti-ERα antibody in normal canine LEC (**A**) and anterior capsulotomy samples from canine patients with naturally occurring cataract (**B**). **B** shows more extracellular matrix surrounding many of the cells, which is typical of LEC that have undergone EMT and make aberrant collagens. Both the nuclei (large arrowheads) and the cytoplasm are immunopositive in the cataractous sample whereas the normal sample has less intense immunostaining in both. Extracellular matrix production is seen in between and above the cataractous LEC (red arrows). Immunostaining results are representative of repeated (three times) experiments. Chromogen is DAB with hematoxylin counterstain. Magnification, 400×. **C**: Canine mammary adenocarcinoma shows nuclear and cytoplasmic immunopositivity in the acini and in some of the surrounding stromal cells. **D**: Canine mammary adenocarcinoma served as a negative control and is representative of all negative control slides used. **E**:  Western blot analysis compares the expression of ERα in normal LEC (three different age groups) and naturally occurring breed-related/presumed-inherited cataractous LEC samples. Normal LEC express ERα less than the cataractous LEC. N=normal canine lens. Controls include canine tumor samples overexpressing ERα (MCT=mast cell tumor, Mel=melanoma).

Immunostaining for ERα was very strong in the nuclei and cytoplasm of naturally occurring cataractous canine LEC and minimal in normal canine LEC (Figure 2A). In addition, cataractous LEC demonstrate extracellular matrix production seen in areas where the capsule appears to be in between and above the LEC (Figure 2B). None of the cells in any sample showed positive immunoreactivity for ERß.

### Western blot analysis of ERα

Western blot analysis with anti-ERα antibody was performed on normal canine LEC from freshly harvested lenses (n=6), anterior capsulotomy samples from canine patients undergoing routine cataract extraction (n=10), and canine tumor controls (mast cell tumor and mammary adenocarcinoma). Expression was present but minimal in the normal LEC when compared to the cataract samples, which showed increased expression of ERα (Figure 2E).

### Quantitative RT-PCR for ERα in normal and cataractous LEC

**Figure 3:**
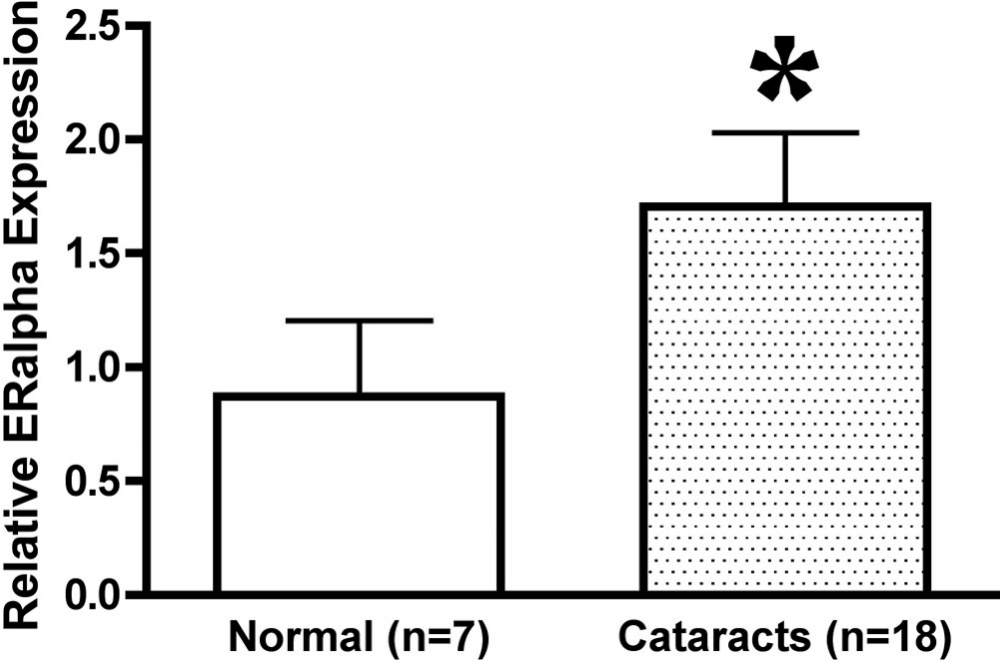
Quantitative RT–PCR for ERα in canine LEC. Data are shown as the gene of interest (*ERα*) divided by the housekeeping gene (*HPRT*). Cataractous LEC had significantly higher *ERα* expression than normal LEC. The asterisk indicates p=0.0423.

Normal canine LEC (n=6) had significantly (p=0.0423) lower expression of *ERα* mRNA than canine cataractous LEC (n=16; Figure 3). Expression of *ERα* relative to *HPRT* expression was approximately two times higher in cataractous LEC.

### Immunoprecipitation of ERα and TERT

**Figure 4:**
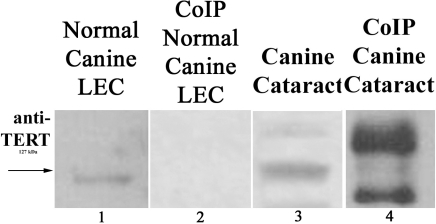
Immunoprecipitation of ERα followed by western blot analysis using anti-TERT antibody. Lane 1 is a non-immunoprecipitated protein sample from normal LEC expressing minimal ERα. Lane 2 shows lack of interaction between ERα and TERT in normal canine LEC. Lane 3 is a non-immunoprecipitated protein sample from cataractous LEC expressing more ERα than normal LEC in Lane 1. Lane 4 demonstrates interaction between ERα and TERT in cataractous LEC. This experiment is representative of two repeated experiments and was performed in reverse twice as well with similar results.

Co-immunoprecipitation showed that ERα is interacting with TERT in cataractous LEC. However, there was no interaction between ERα and TERT seen in normal LEC (Figure 4).

### Phosphorylation of ERα and TERT

**Figure 5:**
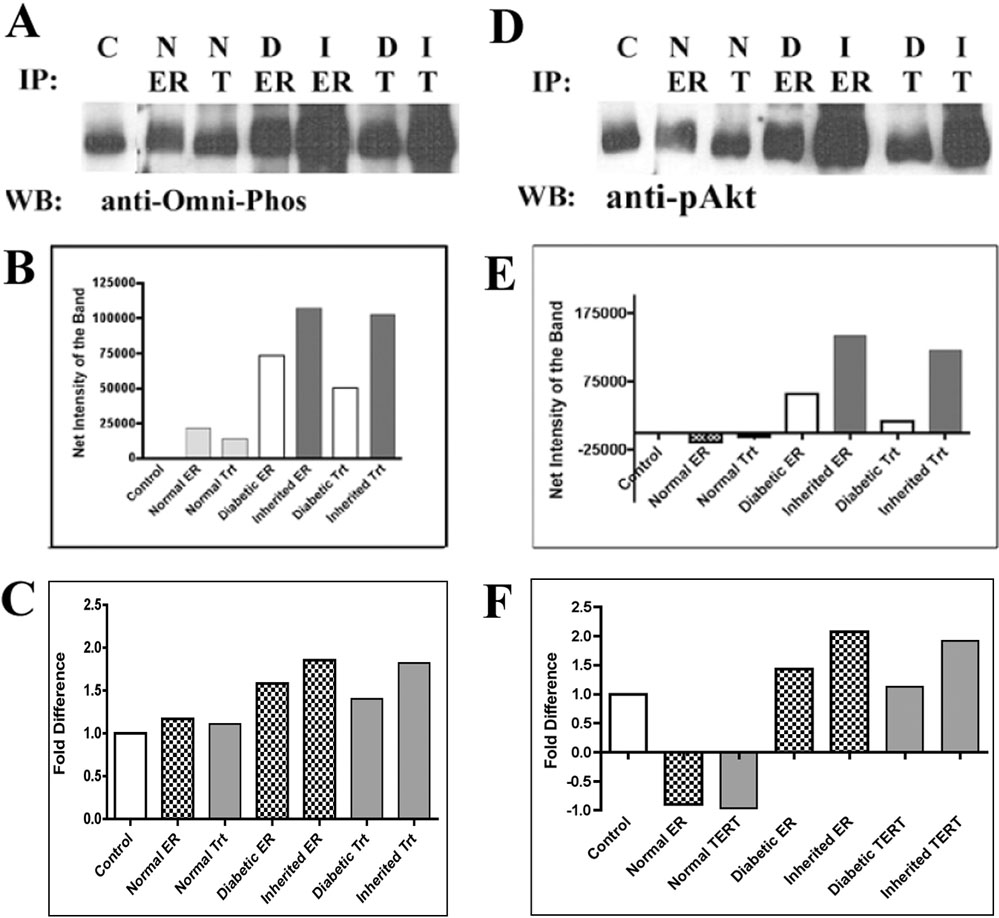
Immunoprecipitation of TERT or ERα from normal and cataractous LEC lysates followed by immunoblotting with anti-Omni-Phos antibody or anti-pAkt antibody. **A**-**C**: Anti-Omni-phos® antibody detected nonspecific phosphorylation of ERα and TERT in both normal and cataractous LEC, though the immunoprecipitated TERT and ERα proteins were shown to be more highly phosphorylated in cataractous than in normal LEC. **D**-**F**: Anti-pAkt antibody revealed that pAkt interacts with ERα and TERT proteins in cataractous LEC but not in normal LEC. These findings imply that phosphorylation of TERT and ERα is Akt-dependent during cataractogenesis and that pAkt is not the kinase involved in normal LEC-telomerase activity. C=control; n=normal LEC; D and I=naturally occurring (diabetic or inherited) canine cataractous LEC; ER or ERα=estrogen receptor alpha; T=TERT. This experiment is representative of two repeated experiments. Statistical analysis was not performed.

Immunoprecipitation with either anti-ERα (ER) or TERT (T) antibodies followed by immunoblotting with either Omni-Phos® or anti-pAkt antibodies was performed (Figure 5). Preliminary phosphorylation evaluation using the Omni-Phos® Phosphorylation Assay Kit identified that ERα and TERT in both diabetic and breed-related cataract samples have more phosphorylation than normal samples. This method also elutes the initial antibody. Therefore, the graph in Figure 5B demonstrates net intensity of each band, that is, the intensity of the control (C, antibody alone) subtracted from the intensities of each band. The graph in Figure 5C demonstrates the fold difference from the control, giving the control a value of 1 and then dividing the intensity signal of each band by the control value.

Since pAkt has been shown to phosphorylate both ERα and TERT, we then performed immunoprecipitation of ERα or TERT with normal and cataractous LEC lysates followed by immunoblotting with the anti-pAkt antibody. The results of this experiment revealed that pAkt interacts with these proteins in cataractous LEC but not in normal LEC (Figure 5D-F). These findings strongly suggest that phosphorylation of TERT and ERα may be Akt-dependent [26] during cataractogenesis and that pAkt is not the kinase involved in normal LEC-telomerase activity.

## Discussion

We previously reported that telomerase activity and its components had a low level of expression in normal canine LEC and significantly increased expression in cataractous LEC [20,27]. TERT is the catalytic subunit of telomerase, and TR is the RNA subunit of telomerase. While TR is expressed at a basal level in all cells, TERT is only expressed in cells that have telomerase activity. Therefore, it is well accepted that TERT expression correlates with telomerase activity [28-31]. TERT is known to function in proliferation and in DNA repair, depending on its location within the cell [32], and it is regulated on all levels, i.e., transcriptionally, translationally, and posttranslationally [33-36]. To find potential candidate proteins involved in interacting with and regulating TERT in LEC, a transcription factor array was used. We found numerous transcription factors with which TERT was interacting and chose to further investigate the ERE and its binding proteins, ERα and ERß. Immunohistochemical analysis revealed that ERß protein was not detectable in normal or cataractous canine LEC and, as such, we focused our efforts on ERα. ERα has been shown to regulate TERT in human ovarian and prostatic cells [26,37,38]. The goal of this study was to determine the expression of ERα in normal and cataractous LEC as well as verify the interaction between ERα and TERT in cataractous LEC. We discovered that there were increased ERα protein and mRNA expressions in cataractous LEC when compared to normal LEC and that ERα and TERT consistently interacted in cataractous LEC. We did not investigate whether the interaction was direct or indirect.

ERα belongs to a superfamily of transcription factors that mediates transcription in a ligand-dependent or a ligand-independent manner. The ligand-dependent manner requires estrogen, and the ligand-independent manner is by means of second messenger signaling mechanisms such as Akt [1,2]. Typically in the absence of estrogen, ERα is complexed with heat shock protein 90 (hsp90), which prevents interaction between ERα and the transcription apparatus [39]. Upon activation, ERα is able to bind estrogen and the receptor undergoes a conformational change that permits binding with co-activators allowing initiation of target gene transcription. We have shown that hsp90 expression is negligible in normal and cataractous LEC, and there is no detectable interaction of hsp90 with ERα or TERT in either normal or cataractous LEC [20]. Therefore, either this interaction is not necessary for ERα to drive downstream regulation or a different co-activator is used by ERα in LEC. It is also possible that so little hsp90 is expressed that an interaction is not detectable by our methods.

ERα has been shown to be present in the rat and human LEC [40-43], and we now demonstrate its expression in canine LEC. We detected low but detectable levels of ERα mRNA and protein in normal canine LEC and increased expression in LEC from canine cataracts. We are presently determining whether this increased expression of ERα is a cause or effect of EMT. A recent report in a breast cancer cell line suggests that there is cross talk among ERα, snail, and the TGF-ß signaling pathway in EMT [44]. All of these proteins have been found in the lens and therefore may be involved in lens EMT. EMT is suggested to be a wound healing response of the LEC to replace abnormal or degenerate LEC [45] and is a feature of LEC in cataracts and in secondary cataract formation (aka posterior capsule opacification, PCO, after-cataract) [46-51]. Cataracts included in this study were late immature, mature, or hypermature cortical cataracts. No nuclear or senile cataracts were included in any experiments. Cortical cataracts in dogs are characterized even at immature stages with EMT of the anterior LEC, resulting in posterior migration of the LEC, subcapsular plaque formation with multiple layering of LEC, and production of aberrant extracellular matrix proteins [22]. In addition, we have found that canine cataract patients develop PCO in 100% of cases when using intraocular lens prostheses that do not have squared edges [52].

ERα mRNA and protein have been found in a variety of ocular cell types in both humans and monkeys [3-5,54-56]. In human retinal pigmented epithelium of both males and females, ERα mRNA and protein expressions have been shown to be regulated in an estrogen-dependent manner [57]. Another study using human eyes found that *ERα* mRNA expression was detected in the retina and retinal pigment epithelium (RPE) of young females but not in males or postmenopausal women. The same study detected ERα in the nonpigmented ciliary epithelium, the iris, and the LEC [43]. Most recently, Cammarata et al. [58] identified ERα in human LEC, suggesting a role for estrogen in lens physiology. As a transcription factor, ERα may be performing a variety of roles in the eye since so many ocular cell types express this protein. Whether estrogen is important in all of these cells has not been evaluated. We are presently attempting to characterize the various aspects of estrogen biosynthesis in LEC in hopes to elucidate why estrogen seems to protect LEC from cataract and whether ERα plays a role in this phenomenon.

Female transgenic mice expressing a dominant negative ERα (i.e., ERα is not able to bind estrogen) develop cortical cataracts, which begin in the equatorial region after puberty and progress with age [59]. These LEC however do not undergo phenotypic changes consistent with EMT or migrate across the posterior capsule (personal communication with Dr. Vicki Davis and evaluation of Dr. Davis’ histological slides by the authors). This suggests that fully functional ERα is important for the EMT that can occur during cataract formation [59]. Although, it is also possible that we have evaluated these lenses too early, i.e., before the LEC undergo EMT.

Estrogen has been shown to enhance ERα and TERT phosphorylation [2,26]. The phosphorylation of ERα results in various functions including DNA binding, recruitment of co-activators, and transcriptional activation [2]. ERα and its co-activators can bind target promoters at the palindromic ERE(s) to stimulate transcription. There are two EREs present on the human TERT promoter (localized in 1 kb of the 5′ flanking region) [37], and two EREs are present on the canine TERT promoter region (GenBank AY833719). Consistent with the transcription factor array, this would support the interaction between ERα and TERT, possibly influenced by estrogen. Unfortunately, we have been unable to culture cataractous canine LEC (i.e., those that have undergone EMT) to evaluate whether ERα is phosphorylated by estrogen in these cells. However, we are in the process of overexpressing ERα in primary cultures of normal LEC in an attempt to evaluate this mechanism.

We were able to preliminarily evaluate the phosphorylation status of both ERα and TERT via co-immunoprecipitation experiments. Using the nonspecific phosphorylation antibodies, we were able to demonstrate that both proteins are phosphorylated in normal and cataractous LEC. Since cataractous LEC have a higher expression of both proteins, it was not surprising that the cataractous LEC had a higher co-expression of the nonspecific kinase mix. Since both proteins are phosphorylated by pAkt in other cell types [60,61], and we had previously found pAkt to have a higher expression in cataractous LEC [62], we chose to include pAkt in these experiments. This is the first study demonstrating that Akt phosphorylates both ERα and TERT in cataractous LEC. Further work is required to identify the kinase that phosphorylates ERα and TERT in normal LEC.

In summary, we have shown that ERα has very low expression in normal canine LEC and increased expression in cataractous LEC, i.e., LEC that have undergone EMT. While we currently do not know the temporal or causal relationship between ERα expression and the process of EMT, we are presently investigating this in our laboratory. In addition, we have determined that TERT, which is also overexpressed in cataractous LEC, is interacting with ERα, and both are phosphorylated by pAkt in cataractous LEC (LEC that have undergone EMT). The role of estrogen in this interaction is unknown. However, we hope that these findings will lead to understanding how estrogen seems to protect LEC against cataractogenesis.
